# Dominant foliar endophytes influence soybean yield and transcriptome

**DOI:** 10.1093/femsec/fiaf053

**Published:** 2025-05-13

**Authors:** Ivan Sosa Marquez, Karla Griesbaum, Lindsay V Clark, Elizabeth A Ainsworth, Natalie Christian, Katy D Heath

**Affiliations:** Department of Microbiology, University of Illinois at Urbana-Champaign, Urbana, IL 61801, United States; Carl R. Woese Institute for Genomic Biology, University of Illinois at Urbana-Champaign, Urbana, IL 61801, United States; Department of Plant Biology, University of Illinois at Urbana-Champaign, Urbana, IL 61801, United States; Carl R. Woese Institute for Genomic Biology, University of Illinois at Urbana-Champaign, Urbana, IL 61801, United States; Roy J. Carver Biotechnology Center, University of Illinois at Urbana-Champaign, Urbana, IL 61801, United States; Department of Plant Biology, University of Illinois at Urbana-Champaign, Urbana, IL 61801, United States; USDA-ARS Global Change and Photosynthesis Research Unit, Urbana, IL 61801, United States; Department of Plant Biology, University of Illinois at Urbana-Champaign, Urbana, IL 61801, United States; Department of Biology, University of Louisville, Louisville, KY 40208, United States; Carl R. Woese Institute for Genomic Biology, University of Illinois at Urbana-Champaign, Urbana, IL 61801, United States; Department of Plant Biology, University of Illinois at Urbana-Champaign, Urbana, IL 61801, United States

**Keywords:** context-dependency, endophytes, Glycine max, *Methylobacterium*, plant–microbe interactions, transcriptomics

## Abstract

Microorganisms associated with plants can affect nutrient and water acquisition, plant defenses, and ecological interactions, with effects on plant growth that range from beneficial to antagonistic. In *Glycine max* (soybean), many studies have examined the soil microbiome and the legume–rhizobium relationship, but little is known about foliar endophytes, their effects on plant biomass and fitness, and how plants respond to their presence. To address these questions, we inoculated *Glycine max* with field-collected isolates of previously isolated, dominant strains of *Methylobacterium* and *Colletotrichum* in either sterile or non-sterile soil. We then used RNAseq to compare the transcriptomic responses of plants to single- and co-inoculation of endophytes. We found that all endophyte treatments increased soybean growth compared to control, but only in sterile soil. These results suggest context-dependency, with endophytes serving as facultative mutualists under stress or nutrient deprivation. Similarly, transcriptomic analyses revealed that soybean defense and stress responses depended on the interaction of endophytes; *Methylobacterium* elicited the strongest response but was modulated by the presence of *Colletotrichum*. Our findings highlight the environmentally dependent effects of co-existing endophytes within soybean leaves.

## Teaser

We explore how microorganism communities associated with plants can affect their host's transcriptome, and change the plant's response to different types of stressors, benefiting the plant and enhancing resilience responses.

## Introduction

As demand for nutritious food around the world increases with our growing population (Saikkonen et al. 2004, Mishra et al. 2021), understanding the myriad effects of microbial communities on plant yield and plant health can help leverage beneficial microbes for plant growth and productivity (Younginger and Friesen 2019, O'Brien et al. 2021). While many environmental factors, such as temperature, precipitation, and nutrient availability, have the potential to alter plant function and productivity (Lobell and Field 2007, Leakey et al. 2009), interactions with microbes may be a source of resilience for plant health and crop yield. Rapid responses of soil microorganisms have even been shown to improve plant fitness in novel environments (*e.g*. Lau and Lennon 2012, de Vries et al. 2023, Ricks and Yannarell 2023)—suggesting that an appropriate microbial community might aid in plant acclimation to changing environments. Thus, understanding how environmental shifts in microbial communities will influence plant growth in various contexts is critical in predicting the effects of plant–microbe symbiosis on plant health.

Harnessing microbes to improve plant health has the potential to make land use more efficient while limiting the need for chemical pesticides that may reduce soil quality, affect human health, and harm surrounding ecosystems (Rani et al. 2021). Though less well-studied compared to the microbial communities residing in roots, foliar (phyllosphere) endophytes are known to cause a wide range of physiological and defense responses in plants. Leaf fungal endophytes, for example, have been reported to improve plant growth under flooded conditions (Adams et al. 2017) and change plant metabolite production, aiding in herbivore defense (Hartley et al. 2015). Foliar fungal endophytes in tropical rainforests can limit pathogen damage to trees (Arnold et al. 2003, Christian et al. 2017) and even alter nitrogen uptake and distribution patterns (Christian et al. 2019). Leaf endophytes may even lead to ladder-up effects on pollinator density, possibly leading to higher yields (Garibaldi et al. 2021).

The transcriptomic responses of hosts to their endophyte communities provide insight into the molecular dynamics of plant–microbe interactions and aid in constructing holistic models of how microbes influence plants both individually and together. Transcriptomic studies can reveal important signaling pathways involved in symbiosis establishment and benefit exchange (Heath et al. 2012, Burghardt et al. 2017); by identifying key genes involved in host–endophyte interactions, we can gain insights into how microbes supplement plant health and productivity in response to biotic and abiotic stressors (Sheibani-Tezerji et al. 2015), as well as understand the recognition and colonization of hosts (Chen et al. 2022). While some studies have described the transcriptomic responses of host leaves to microbial inoculation, resulting in salient gene annotations, most information comes from model host–pathogen symbiosis (*e.g. Arabidopsis– Pseudomonas*) (Zhang et al. 2018). Transcriptomics of host responses to diverse endophytes in both single and mixed inoculations can provide insight into how leaves perceive these symbionts and mediate downstream influences on the fitness of all partners (Christian and Perlin 2024).

*Glycine max* (soybean) is a C3 plant and one of the most widely grown agricultural crops worldwide, with 122.10 million hectares and an annual production of 353 million metric tons (Kumari et al. 2025). Land use for soybeans is increasing due to continued demand for soy-based products for livestock, commercial products, alternative fuel, and non-animal proteins (Alexandratos and Bruinsma 2012). While there is a great deal of data available on root microbial communities in soybean (Meriles et al. 2009, Mendes et al. 2014, Classen et al. 2015) and, more specifically, on the nitrogen-fixing symbiosis between soybean and rhizobia (Han et al. 2020, Li et al. 2020), less is known about the endophytes that reside in the phyllosphere aboveground. In a previous study at SoyFACE (Soybean Free Air CO_2_ Enrichment; Ainsworth and Long 2021), we isolated dominant leaf microbes from soybeans and found that elevated [CO_2_] shifted the assembly of foliar microbial communities; *Methylobacterium* sp. decreased in abundance in elevated (600 ppm) [CO_2_], while an increase in abundance was observed in fungal isolate *Colletotrichum* (Christian et al. 2021). The direct effects of these endophytes on soybean growth and fitness, and thus how shifts in microbial abundance might influence soybean into the future, have remained unclear. Endophytic methylotrophs have been reported to enhance plant growth via both direct and indirect effects on hosts (Kumar et al. 2017). For example, *Methylobacterium mesophilicum* can enhance antioxidant metabolism, which helps protect bacterial cells against damage from free radicals and promotes a successful early interaction between the plant and its rhizobial mutualists (Araújo et al. 2015). Studying these types of additive and non-additive (interactive) effects of microbial community members on host growth is particularly important in multi-player microbial communities, where priority effects, competition, and facilitation play important roles in assembly and host effects (Ayilara et al. 2023).

In this study, we first use inoculation experiments under controlled conditions, both with sterile and non-sterile soils, to study how these dominant foliar endophytes affect whole plant growth and fitness both in single and mixed inoculation treatments. We hypothesized that fungal and bacterial endophytes would have interactive effects and improve soybean growth. In addition, we used RNAseq in soybeans treated with single or mixed inoculation to characterize how leaf transcriptomes respond to these endophytes. We hypothesized that plants would respond to endophyte inoculation, and that they would respond differently to mixed inoculum, compared to single inoculation treatments.

## Materials and methods

The isolates of foliar endophytes we use in these studies originated from the SoyFACE study by Christian et al. (2021), wherein endophytes from field-grown leaves of soybean in either ambient or elevated [CO_2_] were compared using culture-based methods. The most common endophyte was an isolate of *Methylobacterium* sp. (isolate 75.10.b—hereafter “*Methylo”*), followed by *Colletotrichum* fungi. Isolates of *Colletotrichum* (A2.15.12 and A2.10.2) were genetically identical at the ITS locus but had different phenotypes in *in vitro* assays, which is unsurprising given low taxonomic resolution of the ITS (Gazis et al. 2011). Specifically, isolate A2.15.12 (hereafter “*Col1*”) was facilitated by the presence of *Methylo*, whereas isolate A2.10.2 (hereafter “*Col2*”) was antagonized by *Methylo* (Christian et al. 2021).

### Experiment 1: Endophyte inoculation of soybeans grown in sterilized soil

Our experimental design consisted of 30 replicate plants assigned to each of six inoculation treatment groups, for a total of 180 plants. Inoculation treatments were as follows: Control (uninoculated plants), *Col1, Col2*, and *Methylo* in single inoculation, and two co-inoculations consisting of *Methylo* + *Col1* and *Methylo* + *Col2*. Seeds of soybean variety P3IT44E were planted in 6″ pots filled with a 2:1:2 combination of autoclave-sterilized turface: sand: peat (autoclaved on liquid setting for 45 min at 120°C). Each pot was given a small amount of slow-release fertilizer (2.5 g of 13–13–13 Osmocote^®^).

To inoculate plant leaves with fungal endophytes, we transferred *Colletotrichum* hyphae from fungal plates that originated from the initial isolation of endophytes in 2019 to 2% Malt Extract Agar (MEA) plates per isolate, sealed plates with Parafilm, and cultured at room temperature to produce ample spores for plant inoculation (Christian et al. 2021). Hyphal growth was visible after 2 days and filled the plate and produced spores after 10 days. The conidial masses that contain spores appear black in these *Colletotrichum* isolates. We made fungal spore suspensions for inoculation by adding 5 ml of sterilized distilled water to MEA plates and gently scraped them to suspend spores and hyphae. The resulting liquid was poured through sterilized cheesecloth into a 500 ml bottle. We estimated the concentration of spores using a hemocytometer and adjusted both isolates to 1 × 10^6^ cells per ml using PBS (Absher 1973).

We cultured *Methylo* from frozen glycerol stocks stored at -80°C during initial isolation in 2019 by streaking onto solid tryptone-yeast (TY) plates 10 days before inoculation (Oh et al. 1995). After 9 days of growth, single *Methylo* colonies were inoculated into 15 ml Falcon tubes containing liquid TY media and grown in a shaking incubator at 28°C for 24 h before inoculation onto plants to generate adequate inoculum. We combined tubes and diluted the combined liquid cultures with sterilized PBS to an OD600 of 0.1, giving a bacterial concentration of approximately 1.9 × 10^6^ cells/ml as calculated by additional CFU counts. For both the *Methylo* and *Colletotrichum* inocula, we combined suspensions with 0.01% Tween 20 (to promote microbial colonization) into sterilized spray bottles (cleaned with a 10% bleach solution followed by a 70% ethanol solution and then allowed to dry).

We inoculated plants at soybean growth stage V1 (plant has fully developed leaves at unifoliolate node), using 11 sprays (∼10 ml) per leaf per plant of prepared single or mixed suspensions. For mixed inocula (*i.e. Methylo + Col1* and *Methylo + Col2*), single species suspensions were combined in a 1:1 volume ratio. We then immediately covered plants with a 13-gallon plastic bag to increase humidity and encourage infection (Christian et al. 2019). The plants remained covered for 24 h before the bags were removed. We placed plants in a randomized arrangement in the greenhouse, careful to avoid contact between leaves of adjacent plants to limit cross-contamination. We watered soybeans daily for the remainder of the experiment until harvest, being mindful not to wet leaves, again to avoid splashing and thus cross-contamination of endophytes (Saikkonen et al. 2004). Climatic settings were held at 23°C–26°C during the day and 20°C–23°C at night. Plants received 15 h of light from 6:30 a.m. to 9:30 p.m. One week after inoculation, we took preliminary height measurements. We took leaf counts and staked soybeans at growth stage R1 (beginning bloom) after ~4 weeks. We harvested soybeans at growth stage R6 (full seed, 10 weeks post-inoculation) to obtain as many intact leaves as possible (Marcos-Filho et al. 1994). We took data on leaf count, final height, pod count, and weighed all aboveground biomass after drying for 48 h at 50°C. Inspection of roots indicated no nodulation by rhizobia.

### Experiment 2: Endophyte inoculation of soybeans grown in live field soil

To determine if foliar endophytes elicited an effect in microbial conditions more typical of field soils, we performed a second, similar inoculation study but inoculated all pots with soil slurries from SoyFACE. We planted three seeds into each of 160 6″ pots containing LM-6^®^ High porosity mix (Lambert Inc., Rivière-Ouelle, Québec, Canada). Once sprouted, we thinned the soybeans to one plant per pot. One week later, we inoculated plants with slurry from soils that were collected directly from non-experimental soybean fields at SoyFACE. To make the soil slurry, in each of 10 beakers, we submerged 90 g of soil in 300 ml of 0.85% NaCl solution, followed by vortex homogenization and allowing to settle twice (Wendlandt et al. 2022). Each pot received 10 ml of soil slurry supernatant. Inoculation with foliar endophytes took place 4 weeks after planting using the same methods as Experiment 1, with a few exceptions. The concentrations of microbes in all treatment groups were 8 × 10^5^ cells/ml, and soybeans were watered daily via bottom watering. We fertilized soybeans with Osmocote 18–5–8 Plus and commercial fertilizer 15–5–15 cal-mag (300 ppm N) in two treatments pre-flowering to emulate field conditions. All fertilization was stopped 8 weeks before the final harvest. We took measurements of plant height and leaf number 3 days before inoculation, 2 weeks after inoculation, and 7 weeks after inoculation. The final harvest took place 14.5 weeks after initial planting. Data collection was performed as in Experiment 1, with the addition of nodule count.

### Phenotypic data analysis

We conducted all analyses of phenotypic data in Experiments 1 and 2 in RStudio (R 4.1) 2022.02.3+492 “Prairie Trillium” Release for Windows. We used Analysis of Variance (ANOVA) implemented in base R to test for the significance of fungal (*Col1, Col2*, or control) and bacterial (*Methylo* or control) treatments on soybean traits (shoot and root biomass, seed pod number, leaflet number, total height), followed by Tukey tests for means comparison. All figures were made in ggplot2 (Wickham 2024). The CLD (Compact Letter Display) was obtained using the cld function within the emmeans package (Piepho 2004).

### Experiment 3: Soybean transcriptomic responses to endophyte inoculation

Finally, we used transcriptomics to understand the responses of soybean leaves to our endophytic inoculation treatments. We grew 10 plants per treatment under controlled growth chamber conditions with a 23°C “night cycle” (7:00 p.m.–5:00 a.m.) with 0 µmol/m^2^/s light and 28°C “day cycle” (5:00 a.m.–7:00 p.m.) with light at 1100 µmol/ m^2^/s and with humidity set to 50%. We inoculated with a concentration of 10^6^ cells/ml per microbe, following quantification and adjustment methods used in Experiment 1. Plant tissue was harvested 10 days after inoculation by taking 12 punches (total ∼50 mg) from three different leaves with a sterile hole puncher, which were flash frozen in liquid nitrogen before being transferred to a -80°C freezer.

### RNA extraction, sequencing, and data processing

Full details can be found in the Supplemental Methods. In short, we extracted RNA from six samples of frozen plant tissue per treatment (*n* = 36 total) and submitted them to University of Illinois at Urbana Champaign (UIUC) Roy J. Carver Biotechnology Center for library construction and sequencing using NovaSeq^®^ instrumentation. We used the *Glycine max* transcriptome (Glycine_max_v2.1) genome and Annotation 103 from NCBI Reference sequences in Salmon (Patro *et al*. 2017) (version 1.2.1) for quasi-mapping reads to the transcriptome and quantifying transcript abundance, followed by normalization and filtering.

### Analysis of RNAseq and functional annotation

All analyses were conducted on Computer and Network Resource Group's Biocluster high-performance computing resource and R version 4.0.2 (R core team, 2020). Removal of Unwanted Variation (RUV) (Jacob *et al*. 2016) procedure was used to generate latent variables to be used as covariates. The method of Buja and Eyuboglu (1992), implemented in the “sva” package (Leek and Storey 2007), indicated that six latent variables should be added to the model. Negative control genes for RUV were selected based on having *P* > .5 for the effects of endophytes and their interaction using the limma-trend method (Chen et al. 2016), resulting in 3878 negative control genes. Six latent variables optimized to stabilize expression of these 3878 genes were then generated. Differential gene expression analysis was performed with limma-trend using a model including both endophytes and their interaction as independent variables, and latent variables as covariates. Heatmaps were created in pheatmap (Kolde 2019) using scaled logCPM values after adjusting for latent variables, showing clustering of significant genes. Full differential expression analysis using limma-trend method from Experiment 3 revealed that inoculation with *Col1* and its interaction with *Methylo* had minimal impact on the soybean transcriptome (results not shown). Therefore, we present here the results of a simplified model including *Methylo* and *Col2* inoculations only. We used Principal Variance Component Analysis or PVCA (“pvca” R package; Li et al. 2009) to estimate the variance in total gene expression attributable to model effects, incorporating principal components accounting for at least 60% of the variance. To assess Gene Ontology (GO) term enrichment, we used Fisher's exact tests to determine if the number of significant genes (*P*  $ \le $ .05) in GO terms was greater than expected by chance, Julius (2024) was used to assist with GO term data managment.

## Results

### Endophytes increase plant fitness, but only in sterile soil

**Figure 1.**
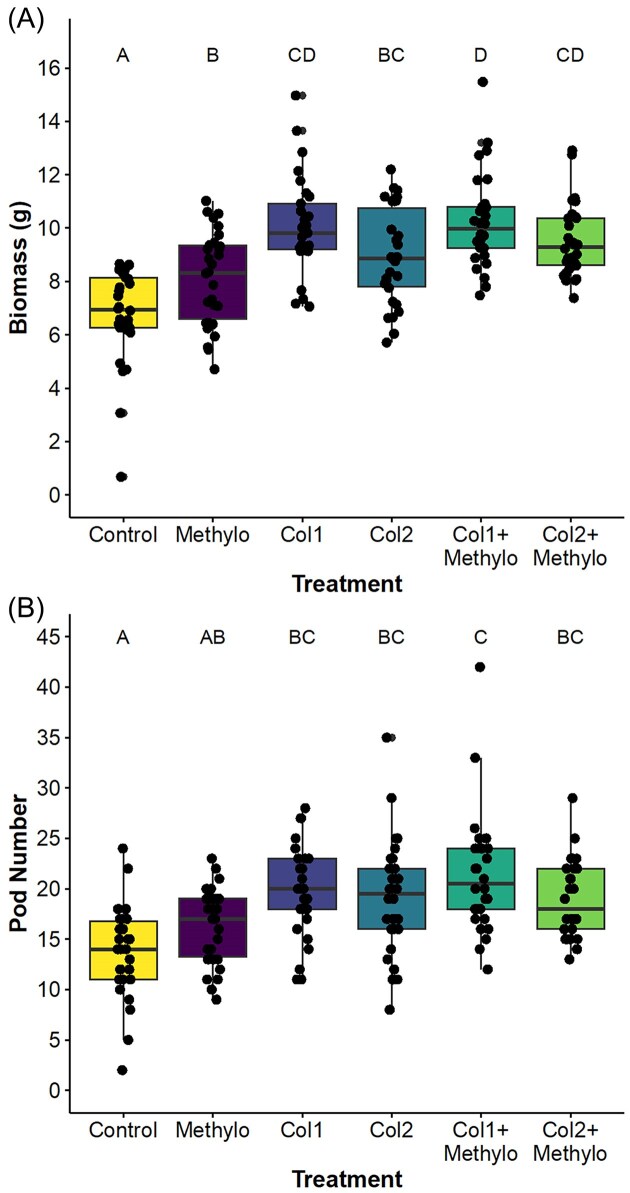
Boxplots showing the effects of inoculation with *Methylobacterium* (*Methylo*) and *Colletotrichum* isolates (*Col1* or *Col2*) either individually or in combination on soybean biomass **(A)** and pod number **(B)** when plants were grown in sterile soil in the greenhouse. Significance of least squares means at the 0.05 level indicated by different letters.

Soybean aboveground biomass and pod number increased in response to endophyte inoculations when plants were grown in sterile soil in the greenhouse (Experiment 1). Inoculation with *Methylo* increased aboveground biomass by 20% compared to controls and resulted in a marginal increase in pod number (Table 1; Fig. 1A and B). Inoculation with *Colletotrichum* had strong effects on biomass and pod number (Table 1); Specifically, isolates *Col1* and *Col2* increased biomass by 49% and 32% and pod number by 42% and 39%, respectively (Fig. 1A and B). We found no significant interactions between *Methylobacterium* and fungal treatments for any traits (Table 1).

**Table 1.** Summary of two-way ANOVA results for the effects of inoculation with *Methylobacterium*, fungi [*Colletotrichum A2.12.15 (Col1)* or *A2.10.2 (Col2)]*, and their interaction on soybean biomass and pod number when soybeans were grown in sterile greenhouse conditions

		Biomass (g)		Pod number	
		den df = 173		den df = 173	
	df	F	*P*-value	*F*	*P*-value
*Methylobacterium*	1	**7.204**	**.0080**	3.36	.0684
*Colletotrichum*	2	**39.019**	**<.0001**	**21.94**	**<.0001**
*Methylo* x *Colletotrichum*	2	1.906	.1518	1.35	.2619

Soybean plants grown in potting mix inoculated with a field soil slurry in the greenhouse (Experiment 2) were much larger (∼7X) and more fecund (∼5.7X) than those grown in sterile soil (Fig. S1). In contrast to the positive effects of bacterial and fungal endophytes on soybean grown in sterile soil, none of the foliar endophytes influenced soybean growth or pod number when pots were inoculated with field soil microbiota (all effects were non-significant; Fig. S1).

### Soybean leaf transcriptomes respond to the interactive effects of endophytes

Next, we used RNAseq to study how leaf transcriptomes respond to the interaction of endophyte inoculations in the absence of soil microbiota. The fungal isolate *Col1* had little effect on soybean transcriptomes (see Methods); thus, we focus here on the results of models including just *Methylo* and *Col2*. The PVCA indicated that 7.6% of the variation in leaf gene expression was attributable to inoculation with *Methylo*, 11.6% was due to *Col2* inoculation, while 20.5% was attributable to the interaction of *Methylo* and *Col2*, indicating strong interaction effects when plants were inoculated with both endophytes. Thirty-four genes responded significantly to the interaction of *Methylo* and *Col2* (Table S1). Many more (298) genes responded to the main effect of *Methylo* inoculation (“*Methylo* genes”), including 26 of the 34 interaction genes (Table S1 and Fig. S2). While the presence of *Col2* modified the responses of soybean genes to *Methylo* inoculation (*i.e*. interaction genes), no genes responded significantly to the main effect of *Col2* inoculation (Table S1).

**Figure 2.**
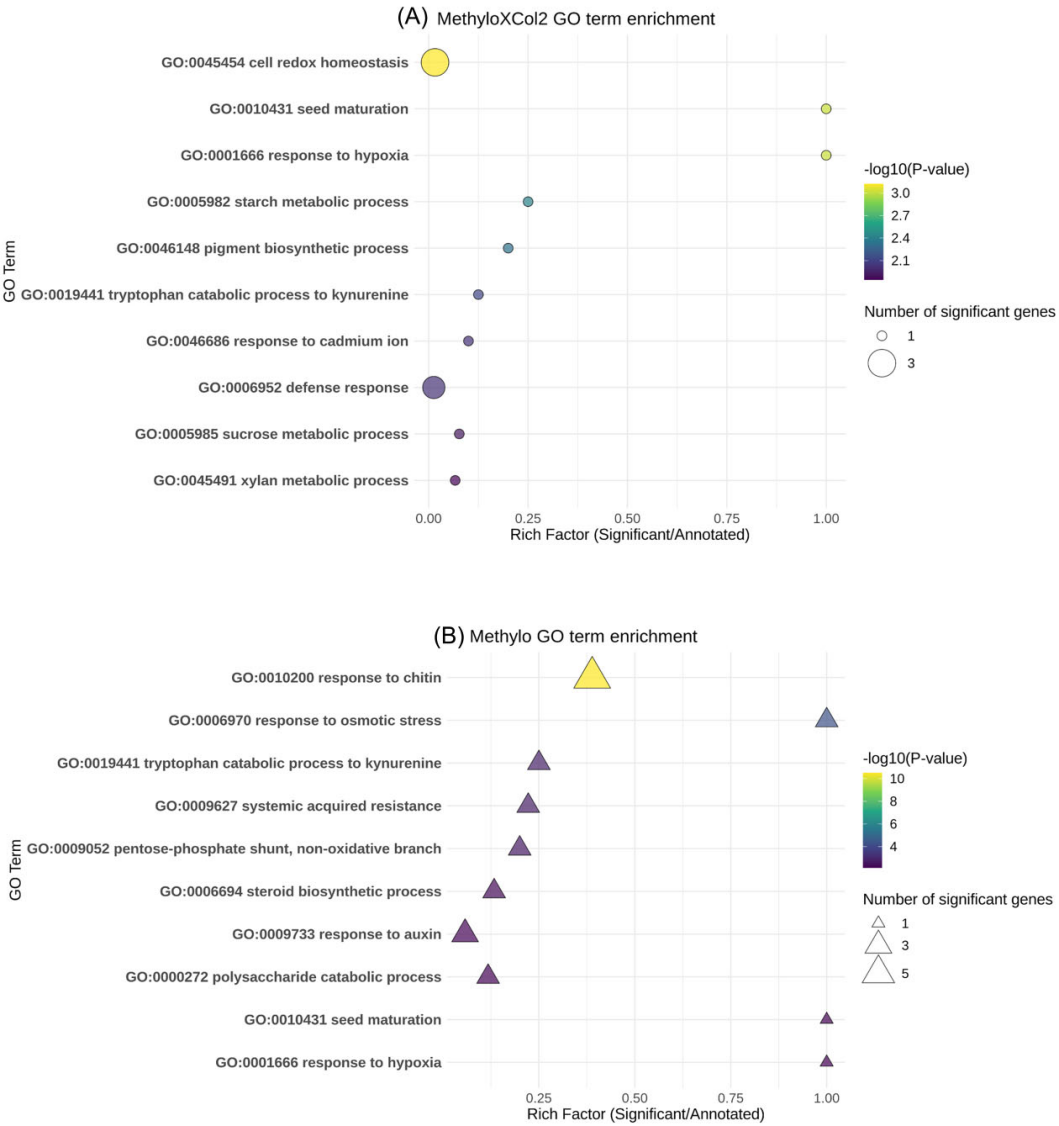
Gene Ontology (GO) terms for genes that responded significantly to (A) *Methylo* x *Col2* interaction, or (B) the main effect of *Methylobacterium*. Top GO terms are ranked by their *P*-values in a Fisher's Exact Test along the *Y*-axis, while the *X-*axis (Rich factor) represents the ratio of significant genes to annotated genes for each GO category. The size of the dots represents the number of significant genes on each GO category.

Next, we used tests of GO enrichment to better understand the functions upregulated when soybeans respond to endophyte inoculation (Tables S2 and S3). Importantly, most differentially regulated genes were unknown and were not assigned GO terms (60% and 66% of interaction-responsive and *Methylo* main effect genes, respectively; Table S1). Nevertheless, GO terms indicating stress and defense were commonly upregulated (Fig. 2; Tables S2 and S3). Three terms (response to hypoxia; GO:0001666, seed maturation; GO:0010431, and tryptophan catabolic process to kynurenine; GO:0019441) were in the top 10 most significant for both gene sets. Additional GO terms related to stress and defense were not in the top 10 for one or both gene sets, but were significant for both (response to chitin; GO:0010200, systemic acquired resistance; GO:0009627, and response to osmotic stress; GO:0006970).

**Figure 3.**
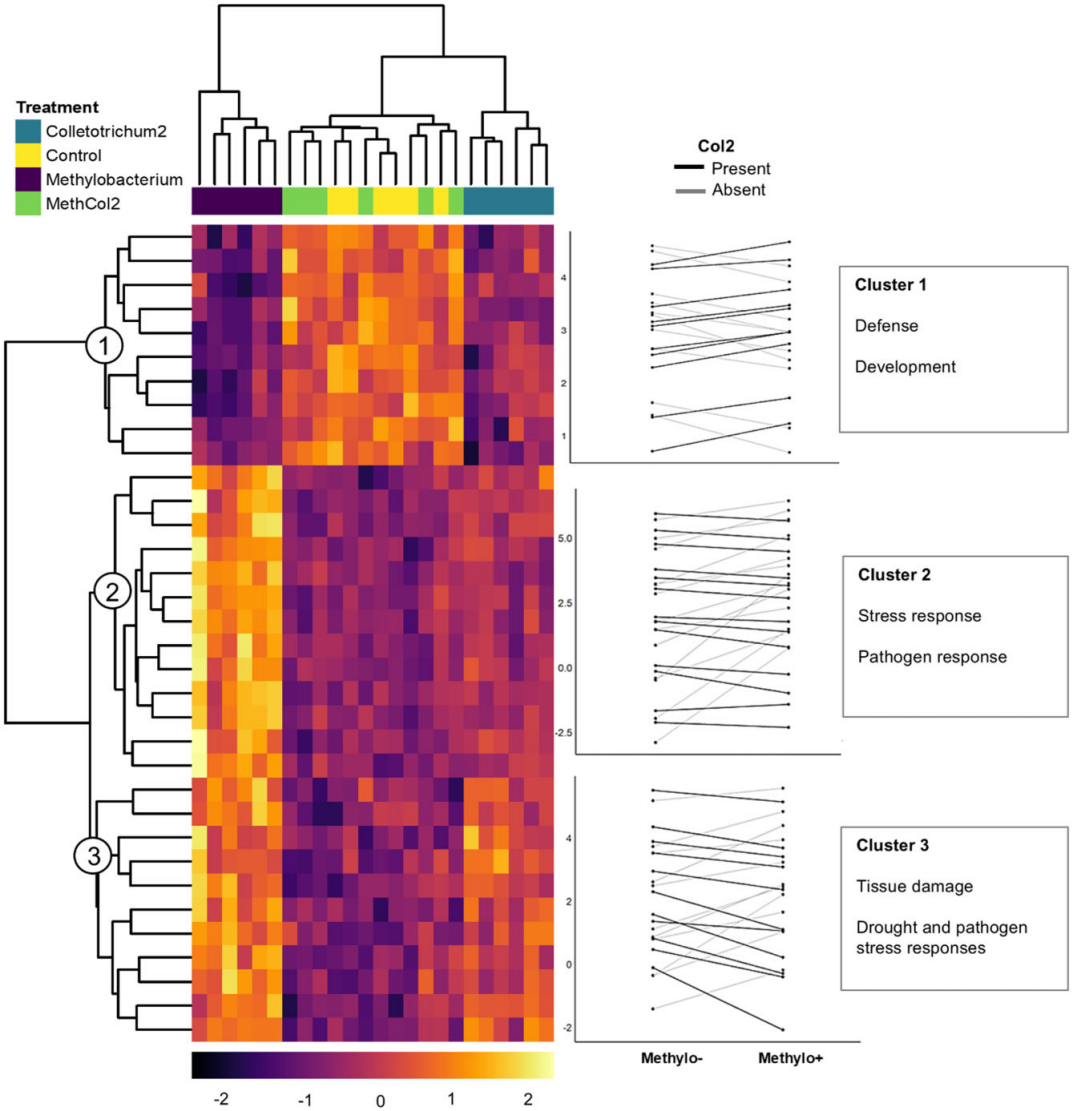
Soybean leaf transcriptomic responses to the interactive effects of inoculation with *Methylobacterium* and *Colletotrichum* (*Col2*) leaf endophytes. Shown is the heatmap of 34 genes that responded significantly to the *Methylo x Col2* interaction; three clusters delineate genes that responded similarly. On the right are reaction norm plots showing that gene expression in each of the three clusters depends on the interaction of both endophytes, along with significant functions indicated by GO analysis.

Inspection of individual interaction-responsive genes across experimental treatments, combined with their annotations, suggests dynamic and context-dependent functional responses of soybean leaves to endophyte inoculations. Overall, responses to *Methylo* inoculation were different, and stronger, when *Col2* was not present, compared to dual inoculation; in the heatmap of interaction-responsive genes, the transcriptomes of plants singly inoculated with *Methylo* clustered together and separately from all other samples, whereas the *Methylo+Col2* samples were most similar to uninoculated controls (Fig. 3). The 34 interaction-responsive genes grouped into three clusters based on their reaction norm patterns (Fig. 3). The first cluster (Cluster 1) features genes associated with defense and normal development (Table S4) and was downregulated in response to single inoculation of either *Methylo* or *Col2*, but expressed similar to baseline (control) under co-inoculation (Fig. 3). Cluster 3 features genes involved in structural maintenance and protein degradation; these genes responded opposite to Cluster 1 and were upregulated in response to single inoculation with either *Methylo* or *Col2* but remained downregulated in co-inoculation (Fig. 3). Finally, genes in Cluster 2 are mostly uncharacterized, but are again consistent with stress and pathogen responses; these genes were upregulated by single inoculation of *Methylo*, compared to all other treatments (Fig. 3).

Of the 298 genes that responded to the main effect of *Methylo*, 195 genes were upregulated compared to controls, while 103 genes were downregulated (Fig. S3). Both up- and downregulated genes featured annotations involved in stress and defense response, gene regulation, transmembrane transport, and lipid metabolism (Table S1). However, upregulated genes also included “response to chitin” (six genes), “proteolysis” (four genes), and “cell redox homeostasis” (four genes). By contrast, downregulated processes included “amino acid transmembrane transport” (three genes) and “auxin-activated signaling pathway” (two genes). In sum, our transcriptome results indicate that plants respond to inoculation with these facultatively beneficial microbes via both known and undescribed signaling and defense mechanisms, and that inoculation with some leaf endophytes (e.g. *Colletotrichum*) can have non-additive, even repressive effects on plant responses to other endophytes (e.g. *Methylo*).

## Discussion

Interactions between plants and their dominant phyllosphere endophytes have important effects on plant nutrition, abiotic stress tolerance, and disease resistance—but occur within diverse microbiomes whose composition and phenotypic effects can change across abiotic and biotic environments. Here, we show that the dominant foliar endophytes of soybean influence plant growth and transcriptomic responses, but that these effects are modulated by the microbial conditions in which they occur. We also show that transcriptomic effects on the plant under sterile soil conditions are dependent on the combination of microbes present in the phyllosphere, with regulatory differences suggesting microbial mediation of genes related to stress, defense, and pathogen response. We discuss the possible explanations for, and implications of, these main results below.

### Phenotypic effects of endophytes are context-dependent

We found that all endophyte treatments used in this study led to increased plant growth in the greenhouse under axenic conditions, but not when plants were inoculated with field soil microbes. Soybeans growing in sterilized soils are missing many root symbionts, including bacterial and fungal mutualists such as rhizobia and mycorrhizal fungi, which are well-known to improve nutrient and water acquisition (Hartnett and Wilson 2002, Kumawat et al. 2019). Our work suggests that the endophytes studied here confer benefits to host plants, but that these effects might be facultative or harder to detect under more natural conditions where plants interact with diverse microbial taxa, which might themselves confer larger fitness benefits, precede endophyte colonization, and/or compete better for shared niches, including phyllosphere. Indeed, Azad and Kaminskyj (2016) found that tomato plants had higher biomass when inoculated with endophytes (compared to control plants), but only under salt or drought stress. A meta-analysis of plant stress mitigation by endophytes showed that endophytes increased biomass accumulation of host plants under drought, nitrogen deficiency, and excessive salinity in forty-two plant species (Rho et al. 2018). Notably, our soybeans grown in sterilized soil did not have access to *Bradyrhizobium* symbionts, which are known to be important for Nitrogen-based mutualism with soybean (Mazur et al. 2024).

Beyond their direct effects on plant growth in microbially depauperate conditions, these endophytes might play other roles in plant health in the field *via* their interactions with other taxa. *Colletotrichum* is a diverse genus and includes members known to inhibit other parasitic fungi from infecting plant leaves (Rodriguez and Redman 2008, Vorholt 2012). Infection of mutualistic or commensal fungi can simply decrease the space available for parasitic fungi (Heydari and Pessarakli 2010, Terhonen et al. 2019) or might have more direct competitive or exclusionary effects on other microbes (Afkhami et al. 2021). These types of context-dependent, multi-player effects make predicting the direct impact of any particular taxon in the microbiome difficult. Simplified experiments under tightly controlled conditions can overestimate the effects of single taxa (Anderson et al. 2013) or alternatively fail to detect important effects because they only occur in some conditions. However, estimating the fitness benefits traded between a host and a particular symbiont in the presence of a diverse community remains a challenge. Novel approaches in microbial ecology have helped enable rapid progress in measuring microbial fitness (Koskella and Vos 2015, Burghardt 2020, Mendoza-Suárez et al. 2021, Burghardt et al. 2022), non-additive effects of complex microbial communities on host traits (Pacheco et al. 2021), and even the implications of various abiotic and biotic treatments on microbial eco-evolution (*e.g*. Lau and Lennon 2012, Koskella and Vos 2015, Ricks and Yannarell 2023).

Microbial communities, and their concomitant effects on host growth and fitness, are likely to be altered under rapid environmental change. In Christian et al. 2021, we found that *Methylobacterium* decreased in abundance while *Colletotrichum* increased in abundance in elevated [CO_2_] environments. The implications of these and other microbial community shifts for soybean production are currently difficult to predict. The health of native plant communities and agricultural crops is predicted to suffer under rising temperatures, frequent drought, and increased ozone due to negative effects on photosynthesis and plant defense (Ayres 1984, Anderegg et al. 2015, Montes et al. 2022). Although many plants experience a fertilization effect of elevated [CO_2_], these and other environmental factors can even negate this effect (Ruiz-Vera et al. 2013, Jin et al. 2018). Nevertheless, endophytes, epiphytes, and soil microbial communities might be more resilient to such changes, and some may be better than others at tolerating novel conditions (Cruz-Martínez et al. 2009, Kashyap et al. 2018). It is possible that host plants might select endophytic fungi that can tolerate higher stresses as plants are exposed to elevated heat and drought conditions and degraded soils (Saikkonen et al. 2004). The immigration of microbial taxa from areas matching the future environmental conditions of an ecosystem might even promote tolerance to climate change factors (Allsup and Lankau 2019). Given their role in plant stress tolerance, predictive models of how changes in soybean endophyte communities will feed back to alter plant health under heat and drought tolerance will be critical (Morsy et al. 2020). Our data highlights that environmental and community dynamics insert a layer of context-dependency between the effects measured in simple manipulative experiments and the implications for plants in real communities.

### Interactive effects of endophytes on the expression of plant defense genes

Using single and co-inoculation combined with RNAseq, we found that *Methylo* and *Col2* had interactive effects on soybean gene expression. Notably, single inoculation with *Methylo* had the strongest effects on host gene expression, but the presence of *Col2* tended to dampen this response. In Christian et al. (2021), community shifts in response to [CO_2_] as well as inhibition of *Col2* growth by *Methylobacterium* on agar plates suggested competition between these two taxa both in the plant leaf and *in vitro*. We note, however, that the assays from Christian et al. (2021) also suggested facilitation of *Col1* by *Methylo*, for which we found little evidence in our phenotypic or expression data. In fact, *Col1* conferred the greatest plant growth benefit in single inoculation, but we found that this isolate had little effect on plant gene expression. Overall, the magnitude and direction of leaf transcriptomic changes *in vivo* are not always predictable from *in vitro* assays, and the magnitude of the plant transcriptomic response does not necessarily scale with the phenotypic effect on plant growth and fitness (Ramírez-Bahena et al. 2024). These findings suggest that interactions among endophytes influence both plant defense mechanisms and gene regulation, shaping how host plants perceive endophytic colonization and driving synergistic effects that alter the leaf transcriptome.

Our results suggest that, at least under sterile conditions, differential regulation of soybean defense signaling occurs in response to the combination of microbes present in the phyllosphere. Bacteria and fungi use a variety of mechanisms to avoid being perceived as pathogens using enzymatic activity, transcriptomic or signaling transduction systems to regulate host receptors, miRNA, kinases, or immune responses (Chen et al. 2022). Our interaction-responsive genes in Cluster 3 suggest that genes involved in plant responses to tissue damage, drought, and microbial and nematode pathogens (including genes annotated as “response to chitin”) are upregulated when either *Methylo* or *Col2* is inoculated individually, but interestingly not when they are inoculated together. Other genes in Cluster 1, involved with defense and responses to environmental stressors like water scarcity or starvation, were regulated in the opposite direction (downregulated when either *Methylo* or *Col2* is present individually, but not together, compared to controls). Based on functional associations derived from soybean as well as plants like *Arabidopsis*, rice, and apple (Table S4), it is difficult to interpret how defense loci in Cluster 3 and Cluster 1 interact within regulatory networks associated with abiotic and biotic stress response, and thus why some are upregulated while others are downregulated. Indeed, even in the best-studied symbioses like legumes and rhizobia, the interplay of defense upregulation and subsequent downregulation is still being worked out (Grundy et al. 2023). Nevertheless, our results indicate that host plants perceive phyllosphere endophytes differently depending on the presence of other endophytes.

Interactive transcriptome effects between *Meth* and *Col2* may arise from mutual inhibition, synergistic signaling enhancing plant colonization, and/or stress regulation. Our *Col2* and *Methylo* strains might simply inhibit each other's growth and thus either's ability to colonize the plant (Christian et al. 2021). However, as studied in beneficial interactions among microbes, bacteria and fungi infecting leaf tissue rely on effectors that enable them to evade plant defense (Wang et al. 2022). Thus, another interesting possibility is that, on their own, either individual endophyte might not have the full set of effectors necessary to attenuate plant defense responses, but together they are effectively complemented by those of the other endophyte; here, the endophytes might benefit from the presence of each other, and thereby potentially benefiting the host when conditions are right. Synergistic effects of co-inoculation of different endophytes in leaves might be like those reported in diverse microbe communities, where pathogenesis-related genes, changes in secondary metabolism, and signaling pathways that depend on the effects caused by more than one microbe help host growth or resilience to stress (*e.g*. Oukala et al. 2021, Liu et al. 2020, Ben Gaied et al. 2024). The involvement of stress-response genes might also suggest that these microbial players might interact with abiotic environmental factors, such as drought, heat, or nutrient deficiency (e.g. Siebers et al. 2015, Dubey et al. 2021, Agunbiade and Babalola 2024).

While *Col2* seems to dampen host transcriptomic responses to colonization, inoculation with *Methylo* resulted in the upregulation of hundreds of soybean genes involved in defense responses. Genes in interaction-responsive Cluster 2 were upregulated in response to *Methylo* but only when *Col2* was absent and were annotated as involved in defense mechanisms, pathogen response, mucigel formation, desiccation, and salt stress responses. Furthermore, many more genes responded significantly to the main effect of *Methylo* but not the main effects of *Col2* or the interaction of *Methylo x Col2*. These observations suggest that inoculation with *Methylo* had much stronger effects on the soybean transcriptome, compared to those of *Col2*. These effects are consistent with many studies showing strong transcriptomic responses to single microbe infection by various bacteria, fungi, and viruses (Wang et al. 2023). *Colletotrichum* might have attenuation mechanisms for these defense and pathogen responses independent from the presence of other microbes (Liang et al. 2018). Although the fitness benefits that microbes could receive from the plant or from each other were not measured here, it is possible that *Methylo* receives bystander benefits by being faced with attenuated plant responses against microbes. Thus, given their abundance in natural phyllosphere communities, *Colletotrichum* species might be very important in diverse microbial communities by alternately facilitating, or competing with, other microbial colonizers—with host effects depending on the identity of would-be colonizers.

## Conclusions

Our findings indicate that foliar endophytes improved soybean growth and fitness, but only when plants were grown in sterile soil, and that the presence of field soil microbes swamped these effects. The complex transcriptional responses of *Glycine max* to inoculation were similarly community-dependent, indicating non-additive effects of these potentially mutualistic bacterial and fungal endophytes in diverse natural communities. Such strain-specific and synergistic responses underscore the need for a more mechanistic and predictive understanding of plant–microbe interactions and thus their implications for crop adaptation and ecological dynamics under a changing climate.

## Supplementary Material

fiaf053_Supplemental_Files

## Data Availability

RNAseq data can be found NCBI's GEO database under BioProject PRJNA823720. All phenotypic data are available in the DRYAD repository accession https://doi.org/10.5061/dryad.wpzgmsbxj.
