# Supplementary material for: Dominant foliar endophytes influence soybean yield and transcriptome
Source: FEMS Microbiol Ecol. 2025 May 13;101(6):fiaf053. doi: 10.1093/femsec/fiaf053 (PMC12089775; doi:10.1093/femsec/fiaf053)
Supplement: fiaf053_Supplemental_Files [file fiaf053_supplemental_files.zip › Supplementary methods 1_Final.docx]

**Supplementary methods 1**

*RNA extraction and sequencing and Library annotation:* Full details can be found in the Supplemental Methods. We extracted RNA from six samples of frozen plant tissue per treatment (n = 36 total) using the QIAGEN® RNAeasy plant Mini Kit with an on-column DNase treatment. For plant library prep and sequencing, samples were submitted to UIUC’s Roy J. Carver biotechnology center. Thirty-six mRNA Libraries were constructed by first selecting polyA RNAs, converting the RNAs to cDNA, performing adaptor ligation, and amplifying. Sequencing was performed using NovaSeq® instrumentation on two 1x100nt lanes, producing over 1 billion reads for the 36 libraries. Reference sequences from the *Glycine max* transcriptome and Annotation 103 from NCBI were utilized for quasi-mapping and count generation. The transcriptome derived from the Glycine_max_v2.1 genome was employed for this purpose. The annotation file facilitated the generation of a transcript-gene mapping table, essential for obtaining gene-level counts. Quality assessment of raw data was conducted by reviewing the FASTQC (version 0.11.8) output from individual samples generated by the sequencing center. A comprehensive report was summarized into a single html report using MultiQC (Ewels *et al*., 2016) version 1.9. All samples exhibited average per-base read quality scores above 30, and no adapter sequences were detected, indicating high-quality reads. Consequently, the trimming step was bypassed, and the data proceeded directly to transcript mapping and quantification.

*Alignment and Gene-Level Quantification:* Salmon (Patro *et al*., 2017) (version 1.2.1) was employed for quasi-mapping reads to the transcriptome and quantifying transcript abundance. The transcriptome was initially indexed using Salmon's decoy-aware method, followed by quasi-mapping with additional arguments to correct for sequence-specific and GC content biases, compute bootstrap transcript abundance estimates, and perform selective alignment. Gene-level counts were estimated based on transcript-level counts utilizing the "bias corrected counts without an offset" method (Soneson *et al*., 2016) from the *tximport* package. The percentage of reads mapped to the transcriptome ranged from 89.9 to 92.9%. Normalization of gene expression levels was performed using the trimmed mean of M values (TMM) normalization method (Robinson & Oshlack, 2010) in the *edgeR* package (Robinson *et al*., 2010) to account for differences in RNA composition. Genes with expression below 0.5 counts per million (cpm) in at least 3 samples were filtered out, resulting in 33,223 genes for further analysis. TMM normalization was re-applied post-filtering.

*RNAseq analyses:* Removal of Unwanted Variation (RUV) (Jacob *et al*., 2016) procedure was used to generate latent variables to be used as covariates. The method of Buja and Eyuboglu (1992), implemented in the “sva” package (Leek & Storey, 2007) indicated that six latent variables should be added to the model. Negative control genes for RUV were selected based on having P > 0.5 for the effects of endophytes and their interaction using the limma-trend method (Chen, *et al.* 2016; Law *et al*. 2014), resulting in 3878 negative control genes. Six latent variables optimized to stabilize expression of these 3878 genes were then generated. Differential gene expression analysis was performed with limma-trend using the with a model comprehending both endophytes and their interaction as independent variables, and latent variables as covariates. Heatmaps were created using scaled logCPM values after adjusting for latent variables, showing clustering of significant genes.

Full differential expression analysis using limma-trend method from Experiment 4 revealed that inoculation with *Col1* and its interaction with *Methylo* had minimal impact on the soybean transcriptome (results not shown). Therefore, we present here the results of a simplified model including *Methylo* and *Col2* inoculations only. We used principal variant component analysis (PVCA1) to estimate the total proportions of variance in gene expression attributable to the aforementioned model effects, using code from the “pvca” R package ( Li *et al*., 2009) with slight modifications to allow logCPM values to be input directly and a threshold of 0.6 (principal components were included to account for at least 60% of the variance in gene expression).

**References**

Buja, A., & Eyuboglu, N. (1992). Remarks on Parallel Analysis. *Multivariate Behavioral Research, 27*(4), 509–540. <https://doi.org/10.1207/S15327906MBR2704_2>

Chen, Y., Lun, A. T. L., & Smyth, G. K. (2016). From reads to genes to pathways: Differential expression analysis of RNA-Seq experiments using Rsubread and the edgeR quasi-likelihood pipeline [version 2; referees: 5 approved]. *F1000Research, 5*, 1438. https://doi.org/10.12688/f1000research.8987.2

Ewels, P., Magnusson, M., Lundin, S., & Käller, M. (2016). MultiQC: Summarize analysis results for multiple tools and samples in a single report. *Bioinformatics (Oxford, England, 32*(19), 3047–3048. <https://doi.org/10.1093/BIOINFORMATICS/BTW354>

Jacob, L., Gagnon-Bartsch, J. A., & Speed, T. P. (2016). Correcting gene expression data when neither the unwanted variation nor the factor of interest are observed. *Biostatistics (Oxford, England, 17*(1), 16–28. <https://doi.org/10.1093/BIOSTATISTICS/KXV026>

Law, C. W., Chen, Y., Shi, W., & Smyth, G. K. (2014). voom: Precision weights unlock linear model analysis tools for RNA-seq read counts. *Genome Biology, 15*(2).

Leek, J. T., & Storey, J. D. (2007). Capturing heterogeneity in gene expression studies by surrogate variable analysis. *PLoS Genetics, 3*(9), 1724–1735. <https://doi.org/10.1371/JOURNAL.PGEN.0030161>

Li, J., Bushel, P. R., Chu, T. M., & Wolfinger, R. D. (2009). Principal variance components analysis: Estimating batch effects in microarray gene expression data. *Batch Effects and Noise in Microarray Experiments: Sources and Solutions*, 141–154. <https://doi.org/10.1002/9780470685983.CH12>

Patro, R., Duggal, G., Love, M. I., Irizarry, R. A., & Kingsford, C. (2017). Salmon provides fast and bias-aware quantification of transcript expression. *Nature Methods, 14*(4), 417–419. <https://doi.org/10.1038/nmeth.4197>

Robinson, M. D., & Oshlack, A. (2010). A scaling normalization method for differential expression analysis of RNA-seq data. *Genome Biology, 11*(3), 1–9. <https://doi.org/10.1186/GB-2010-11-3-R25/FIGURES/3>

Robinson, M. D., McCarthy, D. J., & Smyth, G. K. (2010). edgeR: A Bioconductor package for differential expression analysis of digital gene expression data. *Bioinformatics (Oxford, England, 26*(1), 139–140. <https://doi.org/10.1093/BIOINFORMATICS/BTP616>

Soneson, C., Love, M. I., & Robinson, M. D. (2016). Differential analyses for RNA-seq: Transcript-level estimates improve gene-level inferences. *F1000Research, 4*. <https://doi.org/10.12688/F1000RESEARCH.7563.2/DOI>
